# Impact of local treatment on overall survival of patients with metastatic prostate cancer: systematic review and meta-analysis

**DOI:** 10.1590/S1677-5538.IBJU.2016.0483

**Published:** 2017

**Authors:** Arie Carneiro, Willy Baccaglini, Felipe P.A. Glina, Paulo P. Kayano, Victor M. Nunes, Oren Smaletz, Wanderley Marques Bernardo, Icaro Thiago de Carvalho, Gustavo Caserta Lemos

**Affiliations:** 1 Departamento de Urologia, Hospital Israelita Albert Einstein, SP, Brasil;; 2 Departamento de Urologia, Faculdade de Medicina do ABC, SP, Brasil;; 3 Faculdade de Ciências Médicas, Universidade Metropolitana de Santos, SP, Brasil;; 4 Centro Universitário Lusiada, Faculdade de Ciências Médicas de Santos, SP, Brasil;; 5 Departamento de Oncologia, Hospital Israelita Albert Einstein, São Paulo, Brasil;; 6 Departamento de Radioterapia, Hospital Israelita Albert Einstein, São Paulo, Brasil

**Keywords:** Prostate, Survival, Radiation Oncology, Prostatic Neoplasms

## Abstract

**Context:**

Currently, standard treatment of metastatic prostatic cancer (MPCa) is androgen-deprivation therapy (ADT). Recent studies suggested that local treatment of MPCa is related to increase of survival of those patients, as observed in other tumors.

**Objective:**

To evaluate the impact of local treatment on overall survival and cancer specific survival in 3 and 5 years in patients with MPCa.

**Materials and Methods:**

Systematic review and meta-analysis of population studies published at PubMed, Scielo, Lilacs, Cochrane and EMBASE databases until June 2016. Several large cohorts and Post-Roc studies were included, that evaluated patients with MPCa submitted to local treatment (LT) using radiotherapy (RDT), surgery (RP) or brachytherapy (BCT) or not submitted to local treatment (NLT).

**Results:**

34.338 patients were analyzed in six included papers, 31.653 submitted to NLT and 2.685 to LT. Overall survival in three years was significantly higher in patients submitted to LT versus NLT (64.2% vs. 44.5%; RD 0.19, 95% CI, 0.17-0.21; p<0.00001; I^2^=0%), as well as in five years (51.9% vs. 23.6%; RD 0.30, 95% CI, 0.11-0.49; p<0.00001; I^2^=97%). Sensitive analysis according to type of local treatment showed that surgery (78.2% and 45.0%; RD 0.31, 95% CI, 0.26-0.35; p<0.00001; I^2^=50%) and radiotherapy (60.4% and 44.5%; RD 0.17, 95% CI, 0.12-0.22; p<0.00001; I^2^=67%) presented better outcomes.

**Conclusion:**

LT using RDT, RP or BCT seems to significantly improve overall survival and cancer-specific survival of patients with metastatic prostatic cancer. Prospective and randomized studies must be performed in order to confirm our results.

## INTRODUCTION

Radical prostatectomy (RP) has been reserved for patients with localized disease, and recently, its use was expanded to treat patients with locally advanced disease (1-3). Nowadays, it is discussed the impact of local treatment (LT) also for metastatic prostatic cancer (MPCa) in order to improve survival and time of response to androgen - deprivation therapy (ADT) and systemic progression of the disease (4-8). Standard treatment of patients with MPCa is single ADT, that has a overall survival of 42 months (9).

The treatment of the primary tumor of patients with metastatic disease has been stablished for some types of tumors. Two prospective and randomized studies showed a significant improvement of survival with cytoreductive nephrectomy associated to systemic treatment of patients with renal cell carcinoma (10, 11).

Also, current data show this benefit in relation to other tumors (ovary, gastrointestinal, among others) (12-14). However, until now, there is no study with evidence level 1 that demonstrates such benefit in relation to treatment of primary tumor in patients with MPCa. Recently published retrospective studies showed controversial results in relation to the benefits of LT associated to ADT on overall survival and cancer-specific survival (6, 15-18).

We decided to perform a systematic review and a meta-analysis in order to clarify the role of local treatment on overall survival in 3 and 5 years, as well on cancer-specific survival of patients with MPCa.

## MATERIALS AND METHODS

### Inclusion and Exclusion Criteria

We included case-control studies, big cohorts or clinical trials in English, Portuguese and Spanish, that presented data of patients with metastatic prostate cancer treated with LT (BCT and/or RDT and/or RP) or without LT (NLT) associated or not with ADT. The following aspects were analyzed: overall survival in 3 and 5 years, cancer-specific survival in 3 years and quality of life. Studies that did not separate results of treatment of high risk tumors and metastatic tumors were excluded.

### Databases

Search was performed at MedLine, Lilacs and Embase until June, 26^th^, 2016. The terms included were: “((prostate OR prostatic) AND (cancer OR carcinoma OR tumour OR tumor OR neoplasm) AND (metastatic OR metastasis OR advanced OR “high risk” OR “lymph node” OR nodal)) OR (metastatic prostate cancer OR mPCa) AND (“local therapy” OR cytoreductive OR cytoreduction OR surgery OR prostatectomy OR “radiation therapy” OR radiotherapy OR Brachytherapy) AND (Castration OR Orchiectomy OR “Androgen-deprivation therapy” OR Androgen-deprivation OR “Gonadotropin-Releasing Hormone Agonists” OR “GnRHa treatment” OR “hormone therapy” OR “hormonal therapy” OR “Androgen deprivation” OR “chemohormonal therapy”) OR (Outcomes OR “Perioperative Outcome” OR “Survival Rate” OR “Neoplasm Recurrence”) AND (“prognosis/broad” [Filter] OR “therapy/broad” [Filter] OR “prognosis /narrow” [Filter])”.

### Selection

#### Selection Process

Two authors singly performed the selection of articles according to title. If the theme was adequate to previous stablished criteria or if there was any doubt as the possibility of inclusion, the summary was read. Abstracts were analyzed by three authors and if considered adequate by at least two researchers, the whole article was obtained (19).

#### Checklist

SIGN checklists were used for comparative studies, using cohort and case-control studies.

## Critic evaluation

### Biases

For cohort studies, the analyzed biases included selection biases, performance biases, detection biases, and memory biases. In case-control studies it was analyzed selection biases, detection biases and memory biases.

### Extraction of Results

Selected disclosure was overall survival in 3 and 5 years, and cancer-specific survival in 3 years. Sensitive analysis, when adequate, was performed for patients submitted to LT with or without ADT.

## ANALYSIS

For meta-analysis, RevMan 5.3 software from Cochrane Library was used. For cathegoric variables it was used Cochran-Mantel-Haenszel test and for continuous the reverse variation test. Results were demonstrated by Forest Plot. Heterogeneity was considered acceptable when i^2^<50%, and in those cases it was used a fixed model. Heterogeneity was considered elevated when i^2^≥50%, and in those cases it was used the randomic model. In case of two meta-analysis being analyzed, it was performed the Egger’s test, demonstrated by Funnel Plot. Studies that caused heterogeneity were removed and submitted to a new analysis.

## RESULTS

### Studies Selection

Our search was performed in June, 2016, and identified 19.958 articles, being 9 of grey area (using references of included articles). After the exclusion of 14.994 duplicate articles, 5.014 were selected for detailed analysis of summary, and 5.001 were excluded since they did not fulfill the inclusion criteria. After that, it was performed a detailed analysis of the remaining 28 articles and 16 were excluded due to inclusion criteria and 4 that did not include complete data of one of the targeted population.

**Figure 1:**
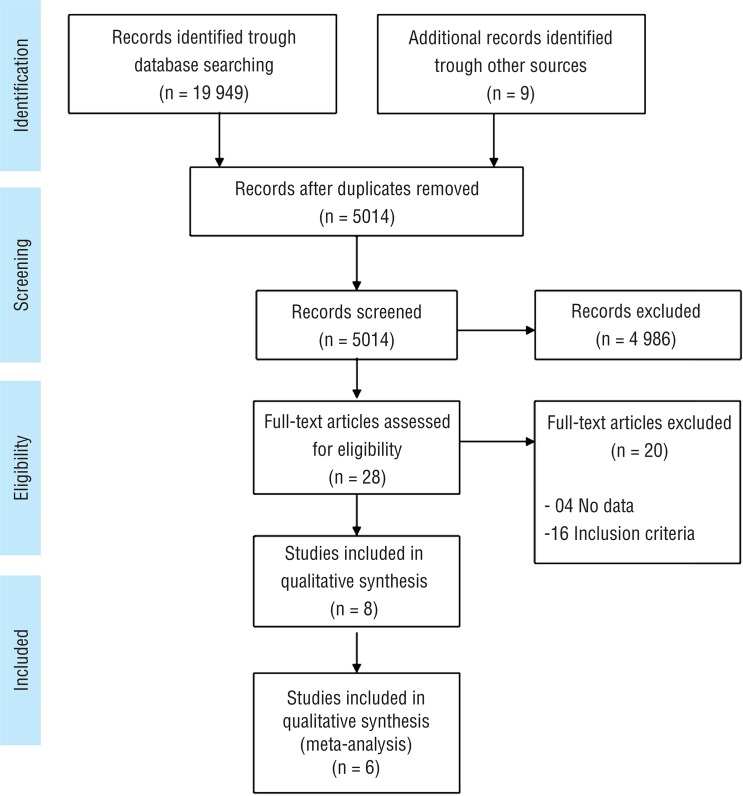
Studies Selection.

In resume, 8 studies were included for systematic review and 6 for meta-analysis, with a total of 34.338 patients (Figure-1).

### Studies characteristics

Seven cohorts were included from the articles (two were not included at meta-analysis since they did not provide adequate data) (20, 21) and one case-control study. No prospective and randomized study was identified (Table-1). For each study we made a detailed analysis of bias (Appendix 1).

**Table 1:** Characteristics of Studies.

Article	Type of Study	Age - I/C (years)	PSA - I/C (mg/dL)	Staging	Intervention	Comparison	Follow-up	N° I	N° C	Outcome
Culp 2014(7)	CR	64/72	Interval	M1a,b,c	RP or BT	NLT	5 years	8185	8185	OS, CSS
Antwi 2014(19)*	CR	Cutoff	Interval	M1a,b,c	RP or BT	NLT	3 years	7858	7858	-
Fossati 2015(20)*	CR	65/71	16/61	M1a,b,c	RP or BT	NLT	3 years	8197	8197	-
Satkunasivam 2015(21)	CR	74/78	246.4/588.4	M1a,b,c	RP or RT	NLT	3 years	4069	4069	OS, CSS
Heidenreich 2015(22)	CC	61/64	135.2/105.9	M1b	RP	NLT	3 years	61	61	OS, CSS
Cho 2016(23)	CR	69	190	M1b,c	RT	NLT	3 years	140	140	OS
Löppenberg 2016(24)	CR	65/69	16/46.7	M1a,b,c	RP, BT or RT	NLT	3 years	38929	15501	OS
Rusthoven 2016(25)	CR	66/69	Interval	-	RT	NLT	5 years	6382	6382	OS

**CR** = cohort retrospective; **M1a** = metastasis in pelvic lymph nodes; **M1b** = bone metastasis; **M1c** = visceral metastasis; **RP** = radical prostatectomy; **RT** = radiotherapy; **BT** = brachytherapy; **NLT** = not submitted to local treatment; **OS** = overall survival; **CSS** = cancer survival specific.

* Studies included just in the sistematic review.

### Synthesis of results

**Figure 2:**
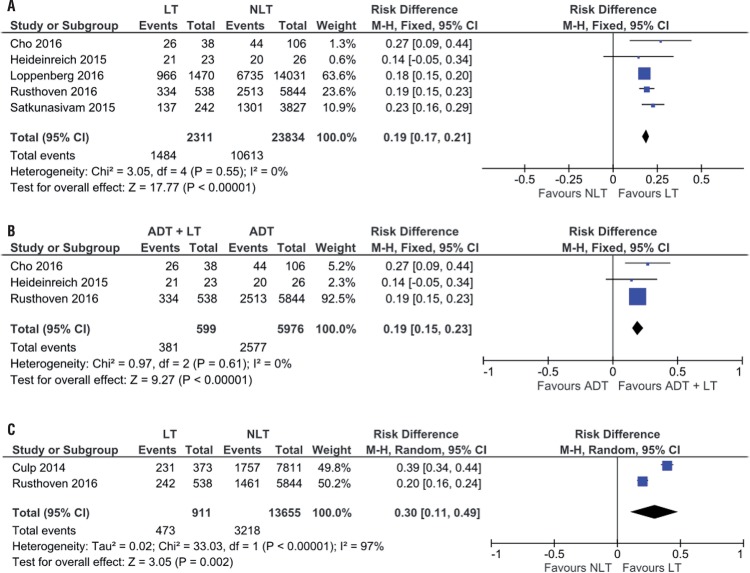
(A) Forest Plot - overall survival in 3 years of patients submitted to LT in relation to those treated with NLT with or without; (B) Forest Plot - sub-analysis of overall survival in 3 years of patients submitted to LT in relation to those treated with NLT with ADT; (C) Forest Plot - overall survival in 5 years of patients submitted to LT in relation to those treated with NLT.

Association Between Overall Survival and Local Treatment in 3 and 5 Years Five studies showed higher overall survival in three years in patients with MPCa submitted to LT in relation to those treated by NLT with or without ADT (64.2% vs. 44.5%; RD 0.19, 95% CI, 0.17-0.21; p<0.00001; I^2^=0%) (Figure-2A). At sub-analysis, when we considered only patients submitted to ADT, we observed the same benefit on overall survival in three years of patients submitted to LT (63.6% vs. 43.1%; RD 0.19, 95% CI, 0.15-0.23; p<0.00001; I^2^=0%) (Figure-2B).

During the analysis of 5-year survival, the results of two studies showed benefits of LT in relation to NLT (51.9% vs. 23.6%; RD 0.30, 95% CI, 0.11-0.49; p<0.00001; I^2^=97%) (Figure-2C).

**Figure 3:**
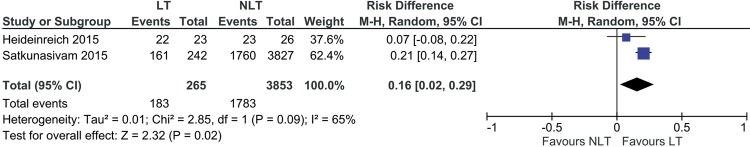
Forest Plot - cancer-specific survival in 5 years of patients submitted to LT in relation to those treated with NLT.

Association of Cancer-specific Survival and Local Treatment in 3 Years Analysis of two studies showed higher cancer-specific survival of patients with MPCa submitted to LT in comparison with NLT (69.1% vs. 46.3%; RD 0.16, 95% CI, 0.02-0.29; p=0.02; I^2^=65%) (Figure-3).

Global and Cancer-specific Survival According to Modality of Local Treatment

**Figure 4:**
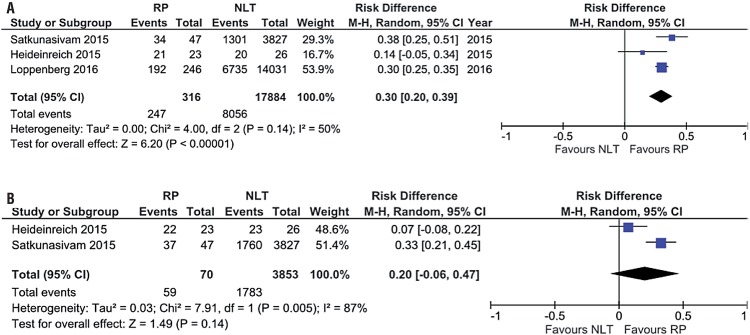
(A) Forest Plot - overall survival in 3 years of patients submitted to LT with RP in relation to NLT group; (B) Forest Plot - cancer-specific survival in 3 years of patients submitted to LT and RP in relation to NLT group.

When we considered local treatment with RP, the results of three studies showed higher overall survival in three years of patients treated with RP and LT in relation to NLT (78.2% and 45.0%; RD 0.30, 95% CI, 0.20-0.39; p<0.00001; I^2^=50%) (Figure-4A).

**Figure 5:**
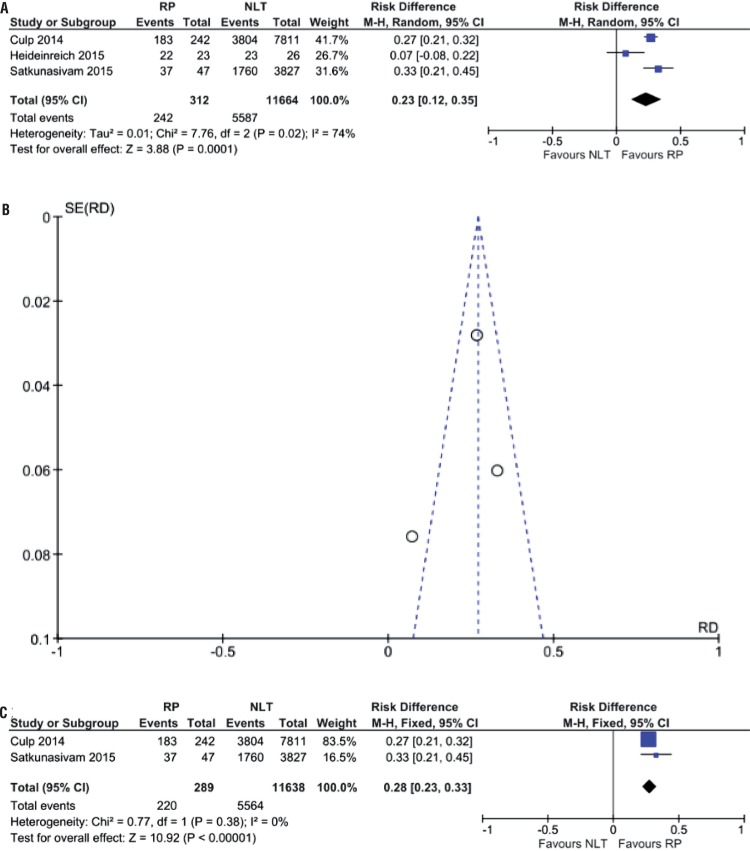
(A) Forest Plot - cancer-specific survival after 3 years of patients submitted to LT and RP in relation to NLT group; (B) Funnel Plot - analysis of sensitivity of cancer-specific survival after 3 years of patients submitted to LT and RP in relation to NLT group; (C) Forest Plot - analysis of sensitivity of cancer-specific survival after 3 years of patients submitted to LT and RP in relation to NLT group.

During analysis of cancer-specific survival in three years it was not observed differences among groups of both included articles (84.3% vs. 46.3%; RD 0.20, 95% CI, -0.06-0.47; p=0.14; I^2^=87%) (Figure-4B). After analysis of three studies, cancer-specific survival was higher in patient submitted to RP (77.6% vs. 47.9%; RD 0.23, 95% CI, 0.12-0.35; p=0.0001; I^2^=74%) (Figure-5A). Again, at sensitivity analysis, LT was favored (76.1% vs. 47.8%; RD 0.28, 95% CI, 0.23-0.33; p<0.00001; I^2^=0%) (Figures 5 B and 5C).

**Figure 6:**
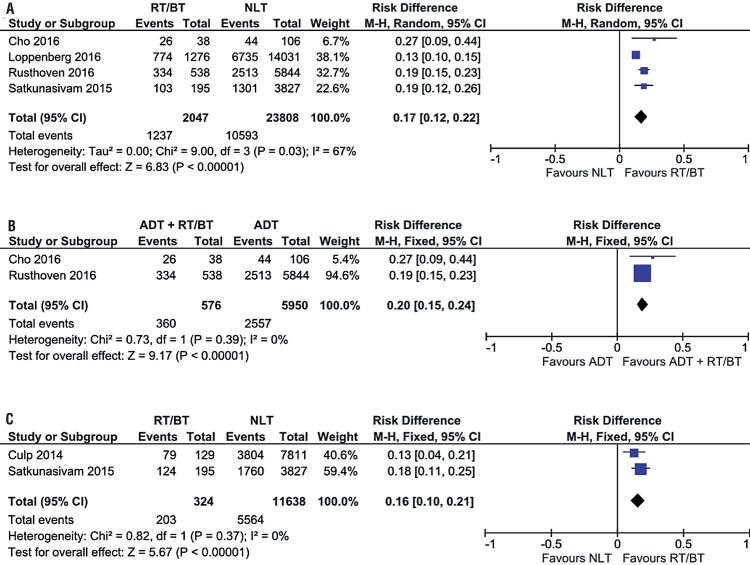
(A) Forest Plot - overall survival in 3 years of patients submitted to LT with RDT or BQT in relation to NLT group with or without ADT; (B) Forest Plot - sub-analysis of overall survival in 3 years of patients submitted to LT in relation to those treated with NLT and ADT; (C) Forest Plot - sub-analysis of cancer-specific survival after 3 years of patients submitted to LT with RDT or BQT in relation to those treated with NLT.

Four studies analyzed patients submitted to radiotherapy (BQT or RDT) and also showed benefit of LT in overall survival in 3 years (60.4% and 44.5%; RD 0.17, 95% CI, 0.12-0.22; p<0.00001; I^2^=67%) (Figure-6A). During sub-analysis, considering only patients submitted to ADT, overall survival in 3 years showed benefit in the group of patients submitted to BQT or RDT (62.5% vs. 43.0%; RD 0.20, 95% CI, 0.15-0.24; p<0.00001; I^2^=0) (Figure-6B). Two studies showed higher cancer-specific survival after 3 years for the first group in relation to control (62.6% vs. 47.8%; RD 0.16, 95% CI, 0.10-0.21; p<0.00001; I^2^=0%) (Figure-6C).

## DISCUSSION

We are living a moment of transition in the profile of patients submitted to RP. In the 90’s, the great majority of those patients were those with low risk prostate cancer. Those with high risk disease were initially submitted to pelvic lymphadenectomy that would stop RP in the presence of a compromised lymph node. In the present, it is recommended to avoid unnecessary treatment of patients with low-risk PCa and for locally advanced disease, even with positive lymph nodes, multimodal treatment with RP and RDT can heal the great majority of patients, and these are the patients who would benefit more with a more radical approach (22).

Until now, there are no level 1 studies that prove the benefit of local treatment of patients with MPCa. Our meta-analysis included big retrospective population studies, among which all five papers (26.145 patients with a median follow-up of 36 months) that evaluated overall survival in 3 years showed benefits with local treatment of patients with MPCa. In 2014, Culp et al. (7), using data collected from SEER, showed higher overall survival in 5 years of patients submitted to RP or BQT in comparison to NLT group. Two other studies extended those benefits for cancer-specific survival of patients with MPCa (23, 24). These series highlight the fact that some men have been treated with RP or RDT even in the absence of clear indication by literature (25).

Frequently, patients submitted to LT were younger, with better clinical conditions and more favorable in relation to GS and PSA at diagnosis, and the NLT group was not homogeneous in relation to the use of ADT. These facts highlight the discussion of the real difference of cancer-specific survival and mortality reached in every respective result. A series published by Rusthovem and cols (26) tried to eliminate these biases making a paired analysis with the same profile of patients in both groups, and also find a positive significance with the addition of local treatment for patients with MPCa.

It is known that the population of patients with MPCa is extremely heterogeneous and it is not possible to extrapolate the indication of local treatment to all patients. In order to try to identify the ideal candidate, Culp et al. (2014) (7) demonstrated an independent association among some variables (age higher than 70 years, Ct4 TNM disease, PSA>20ng/ML, high histological grade and pelvic lymphadenopathy) and an increase of cancer-specific mortality. Antwi et al. (2014) (21) showed that patients with MPCa with low differentiate tumors had a 46% higher risk of death due to all causes and 71% higher in relation to death due to PCa. In relation to the extension of the disease, mortality due to all causes was 52% higher for bone-restricted disease (M1b) and 88% higher for visceral disease (M1c) compared to the single involvement of non-pelvic lymph nodes (M1a). There was an increase of 70% of death due to PCa for M1b disease and a twice higher risk for M1c disease in relation to M1a.

Satkunasivam et al. (2015) (23) showed that advanced age, high levels of PSA, more aggressive high tumor, elevated CCI and bone irradiation less than 6 months of diagnosis are independent factors for the increase of cancer-specific mortality of MPCa patients, and those submitted to RP showed lower mortality when PSA≤20ng/ml. Fossati et al. (2015) (20) found benefits of local treatment in patients with cancer-specific mortality in 3 years predicted to be up to 40%. NNT (number needed to treat) was constant in the interval between 10% and 30%, and rose exponentially when the risk was above 40%. Löppemberg et al. (2016) (27), based on some variables (age, initial PSA, CCI, Gleason Score-Gs and TNM AJCC) also developed a calculus in order to predict global mortality in 3 years of those patients, and concluded that risk over 70% did not add no time to survival with local treatment.

Several models of stratification of metastatic disease have been proposed and all consider visceral and lymph node metastasis important prognostic factors, highlighting the impact of the volume of the metastatic disease. According to SWOG (28), any metastatic lesion other than in the bones, regardless the number of lesions, must be considered high volume disease. Another criteria is the one adopted by the CHARTERED study (29), that considers high volume disease patients with visceral metastasis or >3 bone lesions of extra-axial bone lesion. This study showed that the combined treatment of QT and ADT was benefic in only patients with high volume disease (49 vs. 32.2 months). Cho et al. (30) also showed better prognosis of patients with metastasis restricted to bones in comparison to those with visceral disease. ECOG performance status, local of metastasis, extension of the disease and local therapy with RDT were related to increase of overall survival (ECOG PS 0-1 vs. 2-3, 3-yr OS 65% vs. 23%, p=0.004; M1b vs. other metastasis, 3-yr OS 52% vs. 3%, p=0.005; extension of the disease, single metastasis vs 2-4 metastasis vs. 5 metastasis 3-yr OS 57% vs. 41% vs. 28%, respectively, p=0.007). Therefore, the best candidate to local treatment is the young patient, without significant co-morbidities, and PSA lower than 20ng/ml and low volume metastatic disease (maybe restricted to bones).

Our analysis demonstrates the positive impact of local treatment on survival of patients with MPCa. Literature data show that more than one third of patients without local treatment will present severe local complications due to progression of primary tumor such as: number of hospitalizations, surgical procedures and consequently higher morbidity, with worsening of quality of life of patients (31-33). A case-control study suggests that RP lowers complication rates of urinary tract related to the progression of the disease, while one third of patients of control group presented lower urinary tract obstruction, hematuria or anemia (24). Morbidity and sequelae of local treatment still limit its indication in this scenario of no documented benefit. However, with evolution of technology, including robotic surgery, and more precise modalities of radiotherapy, the morbidity is being significantly reduced.

Surgical treatment of primary tumor is safe in locally advanced PCa (34, 35) and recent papers reproduced these results for metastatic disease. In the study of Cho et al. (2016) (30), 71% of patients treated with RDT received modulated intensity with the aid of helical tomography and none presented severe gastrointestinal or genital-urinary toxicity (grade 3-Radiation Therapy Oncology Group and EORTC criteria). Ten per cent of the RDT group presented hematologic complications grade 3 (Common Terminology Criteria for Adverse Events version 4.0).

However, Sooriakumaran et al. (2016) (25) published a multi-center study with 106 patients with MPCa submitted to RP (open or robotic). In their series, these therapeutic modalities were feasibly and safely performed in selected patients with MPCa, with general and peri-surgical specific complication rates similar to those with localized disease and locally advanced disease. Heidenreich et al. (2015) (24) showed no difference in the follow-up of patients submitted to RP (urinary incontinence and other post-surgical complications) in relation to those with high risk PCa. Complications due to local progression of disease with the necessity of surgical procedures correlated to GS at diagnosis (GS 8-10: 11 of 23, 47.8% vs. GS 7: 0 of 15, p=0.03).

Our work has several limitations. Firstly, our systematic review and meta-analysis were based on retrospective population studies. Secondly, the few studies available in literature are heterogeneous (design, end-points) and they do not allow us to conclude adequately. In third place, not all NLT patients were treated with ADT, but at sub-analysis it was possible to compare LT+ADT vs. ADT alone. And, lastly, local treatment was performed in few patients, that frequently were in better conditions. Anyway, this article presents the best evidence on the subject at the moment. Some big centers already perform prospective and randomized trials in order to evaluate the impact of local treatment on survival. However, it would be very important to also include quality of life analysis objectively with validated question forms (36). Such studies will be fundamental for the evaluation of the real benefit of LT of MPCa and which are the best candidates for such treatment.

## CONCLUSIONS

Local treatment with RDT, RP or BQT seems to contribute significantly to increase overall survival of patients with MPCa. However, prospective and randomized studies are needed to corroborate our data and to identify which patient with MPCa is the ideal candidate for local treatment in a multimode approach.
